# Curcumin Blocks Interleukin-1 Signaling in Chondrosarcoma Cells

**DOI:** 10.1371/journal.pone.0099296

**Published:** 2014-06-05

**Authors:** Thomas Kalinski, Saadettin Sel, Heiko Hütten, Martin Röpke, Albert Roessner, Norbert Nass

**Affiliations:** 1 Department of Pathology, Otto-von-Guericke-University, Magdeburg, Germany; 2 Department of Ophthalmology, University of Heidelberg, Heidelberg, Germany; 3 Department of Hematology and Oncology, Klinikum Braunschweig, Braunschweig, Germany; 4 Department of Orthopedics, Otto-von-Guericke-University, Magdeburg, Germany; Texas Tech University Health Sciences Center, United States of America

## Abstract

Interleukin (IL)-1 signaling plays an important role in inflammatory processes, but also in malignant processes. The essential downstream event in IL-1 signaling is the activation of nuclear factor (NF)-κB, which leads to the expression of several genes that are involved in cell proliferation, invasion, angiogenesis and metastasis, among them VEGF-A. As microenvironment-derived IL-1β is required for invasion and angiogenesis in malignant tumors, also in chondrosarcomas, we investigated IL-1β-induced signal transduction and VEGF-A expression in C3842 and SW1353 chondrosarcoma cells. We additionally performed in vitro angiogenesis assays and NF-κB-related gene expression analyses. Curcumin is a substance which inhibits IL-1 signaling very early by preventing the recruitment of IL-1 receptor associated kinase (IRAK) to the IL-1 receptor. We demonstrate that IL-1 signaling and VEGF-A expression are blocked by Curcumin in chondrosarcoma cells. We further show that Curcumin blocks IL-1β-induced angiogenesis and NF-κB-related gene expression. We suppose that IL-1 blockade is an additional treatment option in chondrosarcoma, either by Curcumin, its derivatives or other IL-1 blocking agents.

## Introduction

Interleukin (IL)-1 signaling through its agonistic proteins IL-1α and IL-1β is involved in inflammatory responses, but also affects malignant processes including tumorigenesis, tumor invasiveness, and tumor-host interactions [1]. As reviewed by Apte et al. [2] IL-1α and IL-1β differ in their subcellular distribution and function in malignant tumors, where IL-1α is mainly active as an intracellular precursor with homeostatic function and as a membrane-bound protein, whereas IL-1β is secreted by macrophages or malignant cells. Microenvironment-derived IL-1β, rather than IL-1α, was found to be required for invasiveness and angiogenesis in different tumor cells [3].

IL-1 signaling starts at IL-1 receptor (IL-1R) with the formation of an active receptor complex by the recruitment of IL-1R-associated kinase (IRAK) to the cytoplasmic domain of IL-1R [4]. Downstream signaling leads to an activation of mitogen-activated protein kinases (MAPKs) and inhibitor of κB (IκB) kinase (IKK), resulting in IκBα phosphorylation, ubiquitination and degradation [5]. Thus, IL-1 signaling leads to an activation of nuclear factor κB (NF-κB) [6]. NF-κB regulates several genes which are involved in tumor cell proliferation, invasion, angiogenesis and metastasis, including VEGF-A [7].

Previously we described the regulation of VEGF-A expression by IL-1β in chondrosarcoma cells [8]. We found that VEGF-A is differentially expressed in conventional chondrosarcomas of different grades with higher levels in high grade tumors, and that VEGF-A expression correlates with the proliferating capillary index [9]. Therefore, we assume that the regulation of IL-1β induced VEGF-A expression is a therapeutic option in chondrosarcomas.

Curcumin is a substance obtained from turmeric (curcuma longa) that modulates several cell signaling pathways [10]. In IL-1 signaling Jurrmann et al. [11] showed that Curcumin inhibits the recruitment of IRAK to IL-1R in murine thymoma cells by modification of IRAK thiols. Thus we asked whether Curcumin is appropriate to block IL-1 signaling in chondrosarcoma cells.

We investigated the effect of Curcumin on IL-1 signaling and VEGF-A expression in C3842 and SW1353 chondrosarcoma cells. We additionally performed in vitro angiogenesis assays and NF-κB-related gene expression analyses. Here we report on these investigations and discuss the therapeutic impact of the results.

## Materials and Methods

### Cell Culture

Human chondrosarcoma cell lines C3842 [12] and SW1353 (purchased from Banca Cellule e Colture in GMP, Genova, Italy) were cultured in RPMI-1640 medium supplemented with 10% fetal calf serum (FCS) and Penicillin/Streptomycin (Biochrom, Berlin, Germany) at 37°C in a humidified atmosphere containing 5% CO_2_.

### IL-1β and Curcumin Treatment

Recombinant human Interleukin-1β (tebu-bio, Offenbach, Germany) was dissolved in sterile water (10 µg/ml). Curcumin (Sigma, Munich, Germany) was dissolved in dimethylsulfoxid (20 mmol/l). 2.5−5×10^5^ cells cultured in serum free medium (approx. 80% confluency) were treated with 10 ng/ml IL-1β for the time indicated (up to 15 min for detection of phospho-IκBα and up to 24 h for detection of VEGF-A). Curcumin was applied in concentrations of up to 20 µmol/l and up to 120 min before treatment with IL-1β, as indicated. Untreated cells were used as controls. N109 renal carcinoma cells were used as positive control for VEGF-A expression.

### Protein Extraction and Quantification

For the detection of VEGF-A the culture medium was collected and concentrated 10-fold using Vivaspin500 centrifugal concentrator (Vivaproducts, Littleton, MA, USA).

For the detection of IκBα and phospho-IκBα the cells were washed with ice-cold PBS and lysed in 400 µl RIPA buffer (50 mM Tris (pH7.5), 5 mM EDTA, 150 mM NaCl, 10 mM K_2_HPO_4_, 10% v/v glycerol, 1% v/v Triton X-100, 0.05% SDS, 1 mM Na_3_VO_4_, 1 mM Na_2_MoO_4_, 20 mM NaF, 0.1 mM PMSF, 20 mM glycero-2-phosphate, and protease inhibitor cocktail (Roche, Mannheim, Germany)) by pipetting. After incubation on ice for 20 min, the lysates were centrifuged (12,000 *g*, 3 min, 4°C). The protein content of the supernatants was measured using the Bio-Rad DC protein assay (Bio-Rad, Munich, Germany) calibrated with bovine serum albumin.

### Western Blot Analysis

50 µl of the concentrated culture medium or 100 µg protein were boiled in Laemmli buffer for 5 min. The samples together with biotinylated protein ladder (Cell Signaling, Danvers, MA, USA) were separated on 13% SDS-PAGE and blotted onto nitrocellulose membranes using a mini transblot cell (BioRad) in blotting buffer (25 mM TRIS, 192 mM glycine, 20% methanol) for 90 min at 100 V. The blots were blocked using Roti-Block (Roth, Karlsruhe, Germany) for 1 h at room temperature. Incubations with rabbit anti-VEGF antibody (1∶1000; Santa Cruz, Santa Cruz, CA, USA), mouse anti-IκBα antibody (1∶1000; Cell Signaling), or mouse anti-phospho-IκBα antibody (1∶1000; Cell Signaling) were performed at 4°C overnight. Secondary incubations with horseradish peroxidase-conjugated goat anti-rabbit or goat anti-mouse antibodies, diluted 1∶50,000, were performed for 1 h at RT. The reactions were visualized by chemiluminescence imaging using using Immobilon chemiluminescent HRP-substrate (Millipore, Schwalbach, Germany) with GeneGnome imaging system (Syngene, Cambridge, UK). After stripping using Restore Westernblot stripping buffer (Thermo, Waltham, MA, USA), subsequent incubations with mouse anti-β-actin antibody (Sigma), diluted 1∶10,000, and secondary incubations with anti mouse antibodies, diluted 1∶50,000, were performed accordingly.

### Immunofluorescence

For immunofluorescence C3842 and SW1353 cells were seeded into 8 well chamberslides (Thermo) and treated with IL-1β (10 ng/ml) for 120 min, and/or Curcumin (20 µmol/l) as described above. Afterwards, the cells were washed twice with cold PBS and immediately fixed with methanol (−20°C) for 10 min and acetone (−20°) for further 5 min. The slides were stored in PBS at 4°C until further use. For immunostaining the slides were blocked in normal goat serum (10%; Vectorlabs, Burlingame, CA, USA) for 60 min at room temperature. Primary antibody directed against the p65 subunit of NF-κB (1∶100; Cell-Signaling) was performed overnight in a humidified chamber at 4°C. After three washes (5 min) with PBS, a secondary antibody labelled with Dylight 549 (1∶1000; Vectorlabs) was applied. The slides were washed with PBS three times for 5 minutes and mounted in DAPI-containing mounting medium (Vectorlabs). For visualisation and photography a Zeiss Axioplan 2 microscope (Zeiss, Oberkochen, Germany) equipped with an ISIS in-situ imaging system (MetaSystems, Altlussheim, Germany) was used.

### Angiogenesis Assay

Human umbilical vein endothelial cells (HUVECs), pre-screened for responsiveness to angiogenic stimuli (Promocell, Heidelberg, Germany) were maintained in endothelial cell growth medium (Promocell). Before angiogenesis assays, HUVECs were subjected to serum starvation for 6 h, detached by the Detachkit (Promocell) and counted. A 96-well plate was prepared on ice by adding 50 µl matrigel (Corning, Wiesbaden, Germany) which was diluted with growth medium to a final concentration of 80%. The gel was allowed to form a matrix at 37°C in the cell culture incubator for 2 h. The cell culture supernatants from C3842 cells were collected after incubation with IL-1β (10 ng/ml) for 24 h, and/or Curcumin (20 µmol/l) as described above. The supernatants were concentrated approximately 50-fold using Amicon Ultra centrifugal filters (Millipore) and diluted 1∶5 with FCS-free cell culture medium. Approximately 50,000 HUVECs were seeded per well together with cell culture supernatants or culture medium with or without 10% FCS. The assays were performed in triplicate. After incubation for 16 h the wells were photographed using a Nikon TS100 inverted microscope and a Coolpix 990 camera (Nikon, Tokyo, Japan). The cellular network was analysed using ImageJ and the Angiogenesis Analyzer application (http://imagej.nih.gov/ij/macros/toolsets/Angiogenesis Analyzer.txt). The total segment length and the number of segments were used for the interpretation of the data.

### RNA-Extraction and RT-PCR

For gene expression analyses, C3842 cells were treated with IL-1β (10 ng/ml) for 6 h, and/or Curcumin (20 µmol/l) as described above. Untreated cells were used as control. RNA was prepared using NucleoSpin RNA II kit (Macherey und Nagel, Düren, Germany) according to the manufacturer’s instructions. 1 µg of total RNA was reverse transcribed using BioScript Moloney Murine Leukaemia Virus (MMLV) Reverse Transcriptase (BioLine, Luckenwalde, Germany) with oligo dT and random hexamer primers (Promega, Madison, WI, USA) in separate reactions. Both reaction products were combined for subsequent analyses. Quantitative PCR was performed using the LC Fast Start DNA Master Plus Sybr Green PCR Mix and a LightCycler 2.0 (Roche, Mannheim, Germany). Human NF-κB Primer Library was purchased from RealTimePrimers (Elkins Park, PA, USA) and thermocycling was performed as suggested by the manufacturers. The relative amount of the targets was calculated according to the ΔΔCt method towards the ribosomal protein 13a, as this gene turned out to be most stable under all conditions applied.

## Results

### Effect of IL-1β on VEGF-A Expression in Chondrosarcoma Cells

**Figure 1:**
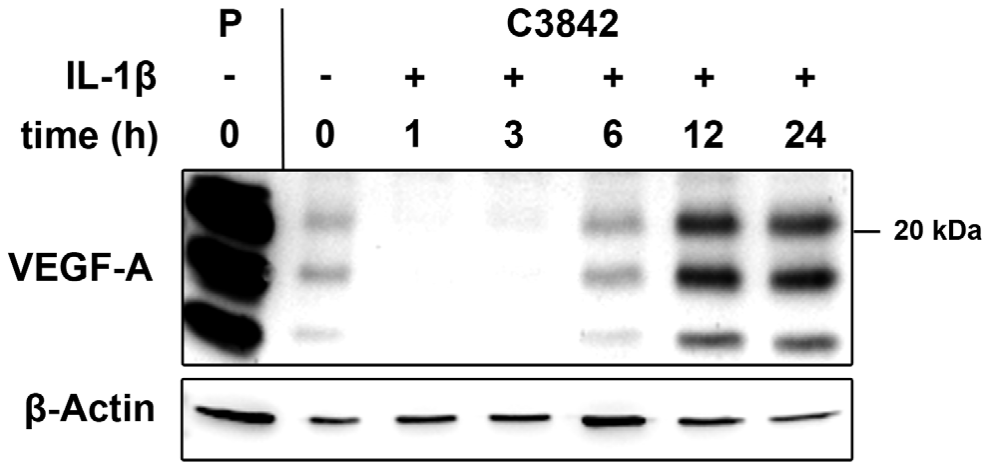
VEGF-A protein expression in C3842 chondrosarcoma cells after treatment with IL-1β (10 ng/ml) for 1 to 24 h, in untreated cells (lane 2) and in VEGF-A overexpressing N109 renal carcinoma cells (lane 1), which served as positive control (P). β-actin is used as loading control.

To analyze the effect of IL-1β on VEGF-A expression we treated chondrosarcoma cells with IL-1β (10 ng/ml). Increased VEGF-A protein levels were detected by western blot in the culture medium of C3842 cells after incubation with IL-1β for at least 6 h or more (Fig. 1). Only basal expression of VEGF-A was detected in untreated cells. A typical pattern of VEGF-A isoforms was observed, which did not change during the incubation. Similar VEGF-A isoforms were also detected in the cell culture medium of VEGF-A overexpressing N109 renal carcinoma cells, which we used as positive control.

### IL-1 Signaling in Chondrosarcoma Cells

**Figure 2:**
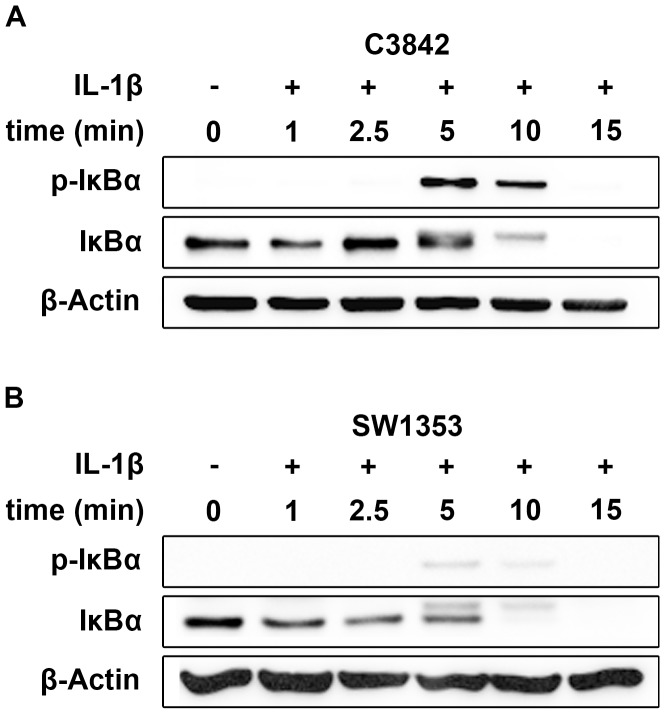
Phosphorylation of IκBα in C3842 (A) and SW1353 (B) chondrosarcoma cells after treatment with IL-1β. The signals disappeared after 15κBα. In untreated cells IκBα is not phosphorylated (lane 1).

For the detection of IL-1 signaling we analyzed the phosphorylation of IκBα by western blot in C3842 cells (Fig. 2A). The IκBα antibody used in this study reacted with IκBα at 39 kDa and phosphorylated IκBα at 40 kDa, whereas the phospho-IκBα antibody was specific for phosphorylated IκBα at 40 kDa. In untreated cells only unphosphorylated IκBα was detected. Phosphorylated IκBα was detected 5 min after incubation with IL-1β. IκBα and phospho-IκBα disappeared after continued incubation with IL-1β. No signals were observed at 15 min due to degradation of phosphorylated IκBα. Similar results were shown for SW1353 cells (Fig.2B).

### Effect of Curcumin on IL-1 Signaling in Chondrosarcoma Cells

**Figure 3:**
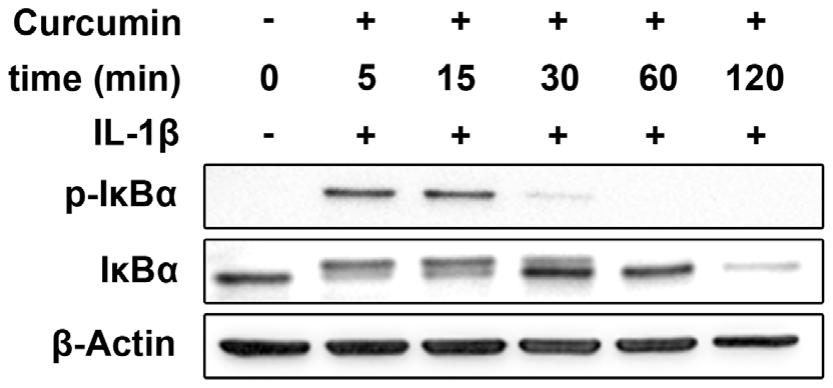
The time of the incubation with Curcumin prior to IL-1β treatment is essential for the inhibitory effect of Curcumin in chondrosarcoma cells. After an incubation of at least 60-1β treatment, Curcumin blocks phosphorylation of IκBα in C3842 chondrosarcoma cells.

To analyze the effect of Curcumin on IL-1 signaling we treated chondrosarcoma cells with variable incubation times and Curcumin concentrations. Curcumin blocked IL-1- induced phosphorylation of IκBα in chondrosarcoma cells depending on the incubation time prior to IL-1β treatment. At least incubation for 60 min with Curcumin (20 µmol/l) was necessary to inhibit IL-1β-induced phosphorylation of IκBα, as shown for C3842 cells (Fig. 3). In subsequent experiments an incubation time of 120 min prior to IL-1β treatment was used.

**Figure 4:**
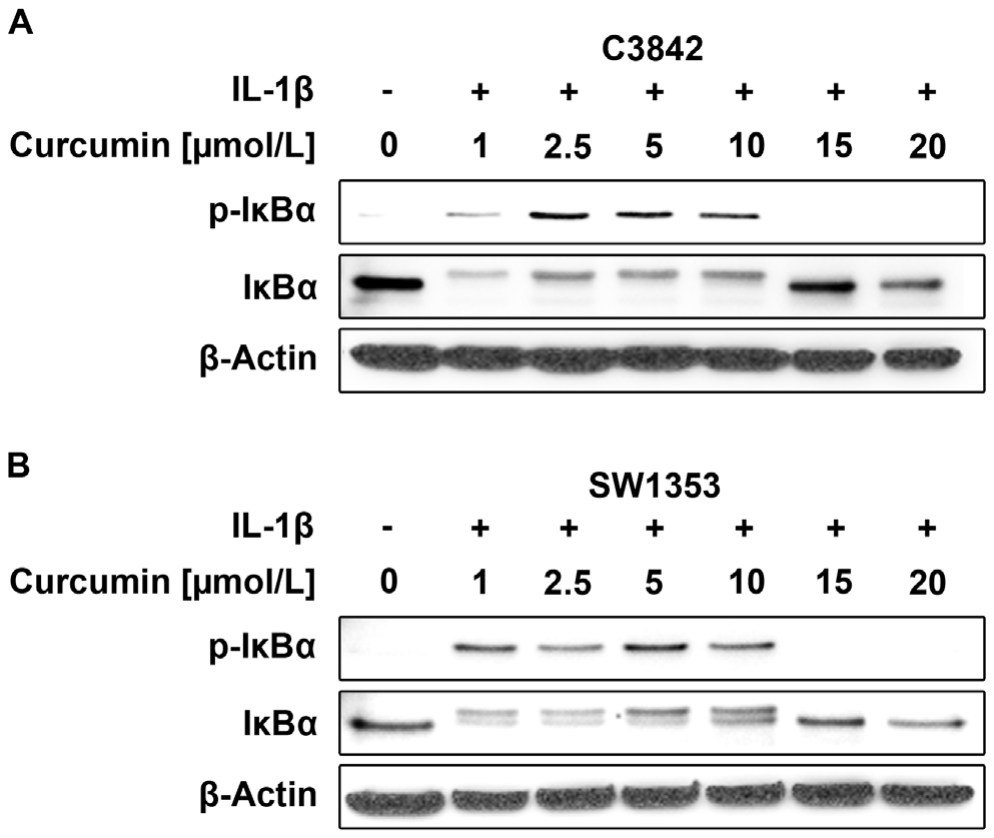
An appropriate concentration of Curcumin is necessary for the inhibitory effect on IL-1 signaling. At least a Curcumin concentration of 15 µmol/l is required to block IκBα phosphorylation in C3842 (A) and SW1353 (B) chondrosarcoma cells.

For C3842 cells and SW1353 cells a Curcumin concentration of at least 15 µmol/l was required to block IκBα phosphorylation (Fig. 4). A Curcumin concentration of 20 µmol/l was used in subsequent experiments.

### Blocking of IL-1β-induced Phosphorylation of IκBα, Nuclear Translocation of NF-κB and VEGF-A Expression by Curcumin

**Figure 5:**
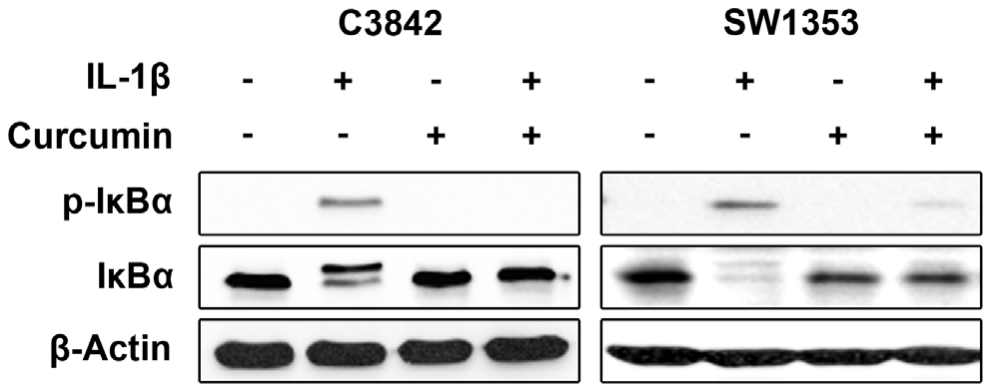
Incubation with Curcumin (20 µmol/l) for 120 min blocks IL-1β-induced phosphorylation of IκBα in C3842 and SW1353 chondrosarcoma cells. Controls with untreated cells, and cells treated either with IL-1β or Curcumin were included.

To demonstrate blocking of IL-1β induced phosphorylation of IκBα we treated C3842 and SW1353 cells with IL-1β after incubation with Curcumin (20 µmol/l) for 120 min. Controls with untreated cells, and cells treated either with IL-1β or Curcumin were included. As expected, Curcumin efficiently blocked IL-1β-induced phosphorylation of IκBα (Fig. 5). No IκBα phosphorylation was detected in cells treated with Curcumin or in untreated cells.

**Figure 6:**
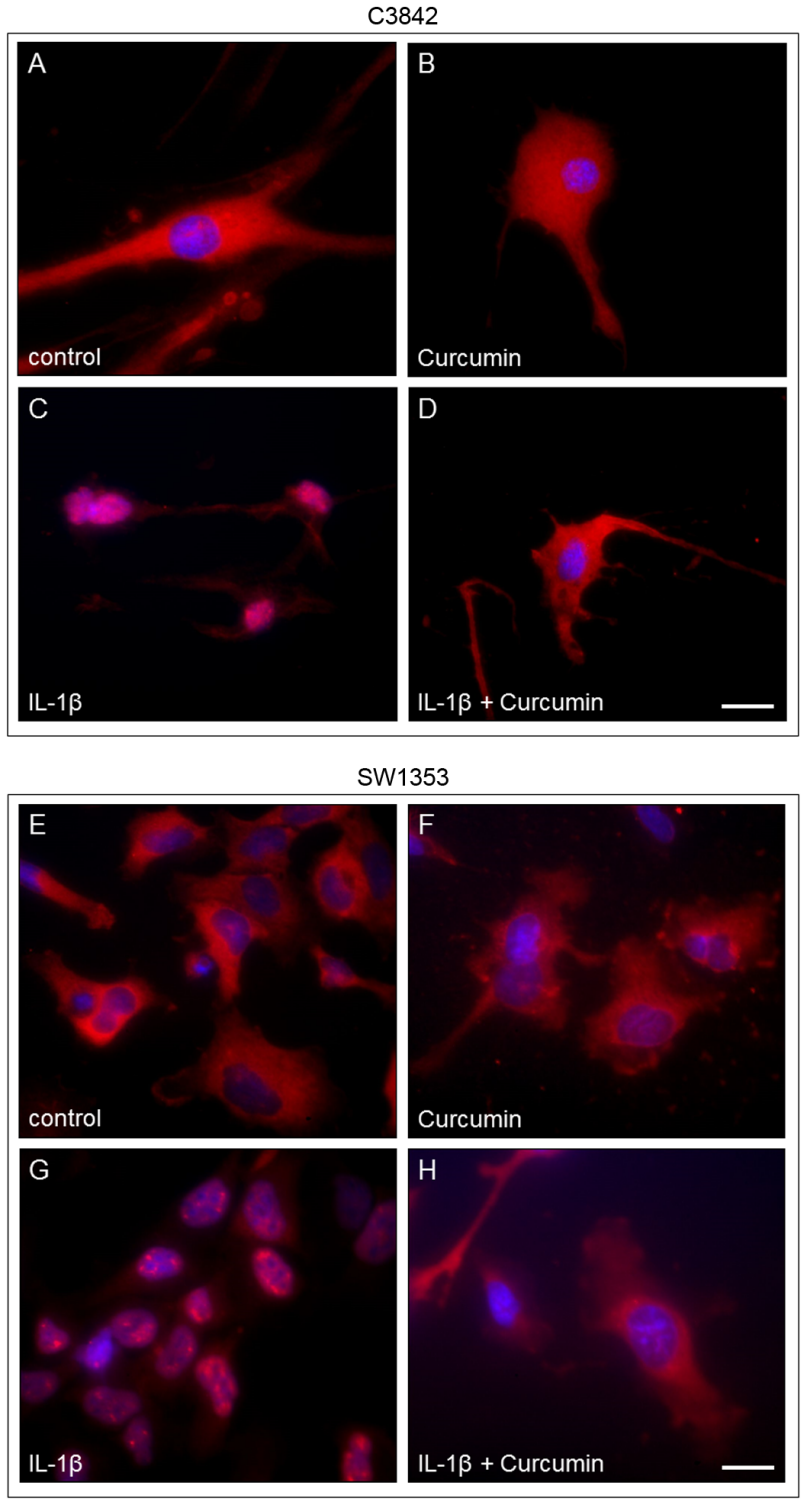
Detection of IL-1β-induced nuclear translocation of NF-κB by immunofluorescence in C3842 cells (A–D) and SW1353 cells (E-H). In untreated cells and cells treated with Curcumin or Curcumin+IL-1β, NF-κB was detected in the cytoplasm, not in the nucleus (Figure 6). In cells treated with IL-1β nuclear translocation of NF-κB was evident.

To show nuclear translocation of NF-κB in chondrosarcoma cells we used immunofluorescence. In untreated cells and cells treated with Curcumin (20 µmol/l) the p65 subunit of NF-κB was detected in the cytoplasm, not in the nucleus (Fig. 6). In chondrosarcoma cells treated with IL-1β nuclear translocation of NF-κB was evident. Curcumin blocked nuclear translocation of NF-κB by IL-1β in C3842 and SW1353 cells.

**Figure 7:**
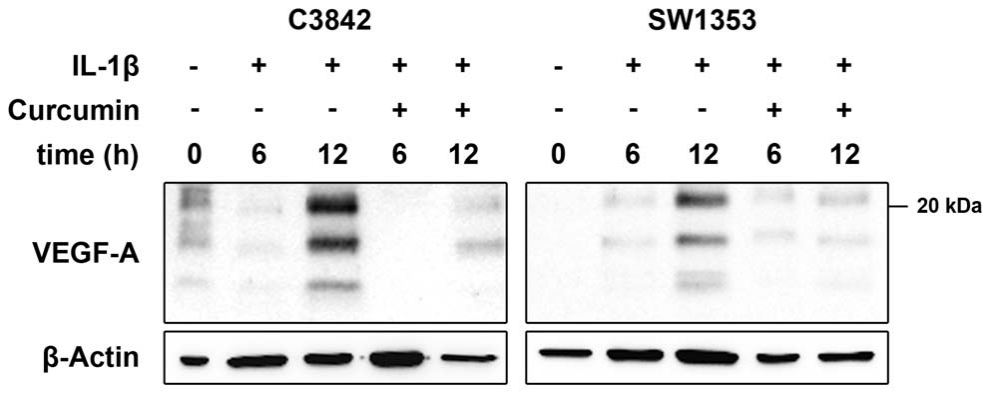
Effect of Curcumin on VEGF-A expression. IL-1β-induced VEGF-A expression is blocked in C3842 and SW1353 chondrosarcoma cells after incubation with Curcumin.

To demonstrate blocking of IL-1β-induced VEGF-A expression by Curcumin we used western blot. Disregarding low basal VEGF-A expression Curcumin blocked IL-1β induced VEGF-A expression in C3842 and SW1353 cells (Fig. 7).

### Blocking of IL-1β-induced Angiogenesis by Curcumin

**Figure 8:**
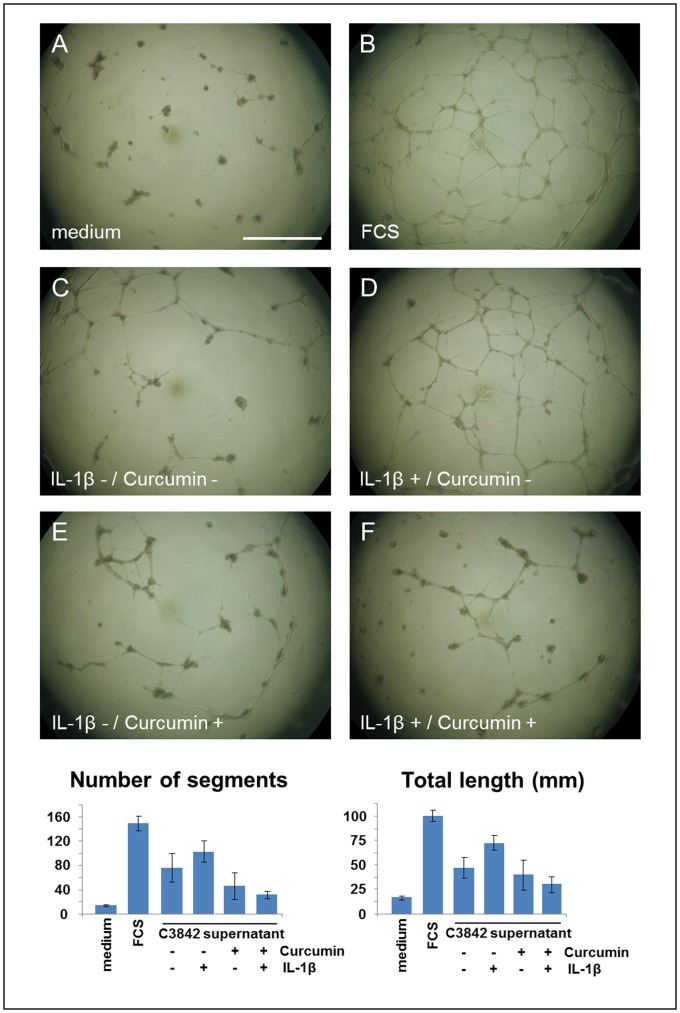
Angiogenesis assay with quantitative image analysis. Cell culture supernatants from C3842 cells treated with IL-1β led to increased number and length of microvessel segments (D). Treatment with Curcumin blocked IL-1β-induced tube formation (F). Controls with supernatants from untreated cells (C) and cells treated with Curcumin (E) were included. Control with FCS-free medium (A). Control with medium supplemented with FCCS (B).

To analyze the effect of Curcumin on angiogenesis we used an in vitro tube formation assay and quantitative image analysis. Cell culture supernatants from C3842 cells treated with IL-1β lead to increased number and length of microvessel segments. Treatment with Curcumin blocked IL-1β-induced tube formation as shown in Fig. 8. Controls with supernatants from untreated cells and cells treated with Curcumin were included. The highest number and length of microvessel segments was detected in HUVECs treated with medium supplemented with FCS. The lowest number and length of microvessel segments was detected in HUVECs treated with FCS-free medium.

### Gene Regulation in Response to IL-1β and Curcumin

To study the impact of IL-1β and Curcumin on NF-κB related gene regulation in more detail, we screened C3842 cells for IL-1β and Curcumin effects on the expression of 88 genes that are related to the NF-κB pathway. The PCR primer array contained NF-κB regulated genes as well as NF-κB signaling components and a set of 8 house-keeping genes as controls. We first tested for the most stable house-keeping gene and decided to use the ribosomal protein 13a (RPL13a) as reference gene as it was unregulated in response to IL-1β and Curcumin. From the other genes we could obtain good amplifications with consistent melting curves in RT qPCR for 72 amplicons. The results are summarized in table 1. As compared to untreated cells, 27 of the analyzed genes were induced by IL-1β more than 2-fold, such as Colony stimulating factor 2 (CSF2), IL-1β, Intercellular adhesion molecule 1 (ICAM1), Chemokine (C-C motif) ligand 2 (CCL2) and IL-6, and only 4 were repressed by more than a factor of 2, such as Inhibitor of kappa light polypeptide gene enhancer in B-cells, kinase gamma (IKBKG), Toll-like receptor 3 (TLR3) or Fas ligand (FASLG). 41 genes were not regulated beyond the thresholds, among them the putative house-keeping genes.

**Table 1:** Relative expression of genes associated with NF-κB. RNA was extracted from C3842 cells treated with IL-1β (10 ng/ml), Curcumin (20 µmol/l) or both.

Gene	IL-1β	Curcumin	IL-1β+Curcumin	Curcumin effect
CSF2 Colony stimulating factor 2 (granulocyte-macrophage)	**27175.14**	**50.56**	**7696.57**	*0.28*
IL1B Interleukin 1, beta	**3258.52**	0.53	**7.62**	*0.00*
ICAM1 Intercellular adhesion molecule 1 (CD54)	**666.29**	**3.34**	**32.22**	*0.05*
CCL2 Chemokine (C-C motif) ligand 2	**127.12**	*0.46*	1.43	*0.01*
IL6 Interleukin 6 (interferon, beta 2)	**99.73**	*0.27*	1.89	*0.02*
CD40 CD40 molecule, TNF receptor superfamily member 5	**66.72**	**2.31**	1.97	*0.03*
CSF1 Colony stimulating factor 1 (macrophage)	**46.85**	**2.17**	**3.07**	*0.07*
TNFRSF1A Tumor necrosis factor receptor superfamily, member 1A	**21.41**	*0.12*	0.54	*0.03*
IL1A Interleukin 1, alpha	**16.45**	1.59	1.42	*0.09*
TNFSF15 Tumor necrosis factor (ligand) superfamily, member 15	**16.11**	*0.48*	0.69	*0.04*
TNFAIP3 Tumor necrosis factor, alpha-induced protein 3	**12.30**	*0.19*	**8.40**	0.68
RELB V-rel reticuloendotheliosis viral oncogene homolog B	**10.48**	**8.28**	**4.29**	*0.41*
IRAK2 Interleukin-1 receptor-associated kinase 2	**9.85**	*0.40*	3.71	*0.38*
IRAK1 Interleukin-1 receptor-associated kinase 1	**9.19**	**2.14**	1.88	*0.20*
NFKBIA Nuclear factor of kappa light polypeptide gene enhancer in B-cells inhibitor, alpha	**7.67**	*0.19*	10.27	1.34
IKBKE Inhibitor of kappa light polypeptide gene enhancer in B-cells, kinase epsilon	**6.32**	0.81	*0.46*	*0.07*
NFKB1 Nuclear factor of kappa light polypeptide gene enhancer in B-cells 1 (p105)	**5.86**	*0.24*	1.95	*0.33*
HTR2B 5-hydroxytryptamine (serotonin) receptor 2B	**5.03**	0.70	13.36	**2.66**
ELK1 ELK1, member of ETS oncogene family	**3.73**	1.27	1.16	*0.31*
FOS V-fos FBJ murine osteosarcoma viral oncogene	**3.41**	**17.15**	4.99	1.46
CFLAR CASP8 and FADD-like apoptosis regulator	**3.29**	*0.49*	1.06	*0.32*
IL12A Interleukin 12A	**2.83**	1.19	1.36	*0.48*
RELA V-rel reticuloendotheliosis viral oncogene homolog A	**2.60**	0.83	0.92	*0.35*
TNFRSF10B Tumor necrosis factor receptor superfamily, member 10b	**2.38**	0.93	0.92	*0.39*
BIRC2 Baculoviral IAP repeat-containing 2	**2.28**	0.61	0.71	*0.31*
IKBKB Inhibitor of kappa light polypeptide gene enhancer in B-cells, kinase beta	**2.23**	0.68	0.58	*0.26*
TNFRSF10A Tumor necrosis factor receptor superfamily, member 10a	**2.10**	1.22	1.25	0.59
*GAPD Glyceraldehyde phosphate dehydrogenase*	1.95	1.83	1.95	1.00
TLR4 Toll-like receptor 4	1.95	*0.35*	*0.27*	*0.14*
REL V-rel reticuloendotheliosis viral oncogene homolog (avian)	1.84	0.57	0.95	0.52
CASP1 Caspase 1, apoptosis-related cysteine peptidase	1.84	*0.36*	*0.36*	*0.19*
IFNA1 Interferon, alpha 1	1.74	1.28	1.22	0.70
RHOA Ras homolog gene family, member A	1.72	1.52	1.67	0.97
BCL2L1 BCL2-like 1	1.68	**4.35**	1.27	0.76
TICAM2 Transmembrane emp24 protein transport domain containing 7	1.55	1.00	0.27	0.17
RAF1 V-raf-1 murine leukemia viral oncogene homolog 1	1.54	1.02	0.99	0.64
STAT1 Signal transducer and activator of transcription 1	1.54	1.10	1.27	0.83
CASP8 Caspase 8, apoptosis-related cysteine peptidase	1.52	*0.44*	0.53	*0.35*
EDARADD EDAR-associated death domain	1.47	1.67	1.48	1.01
EDG2 Lysophosphatidic acid receptor 1	1.47	1.27	1.15	0.78
TMED4 Transmembrane emp24 protein transport domain containing 4	1.45	1.47	3.12	2.14
RIPK1 Receptor (TNFRSF)-interacting serine-threonine kinase 1	1.35	0.75	*0.16*	*0.12*
TBK1 TANK binding kinase 1	1.28	*0.17*	*0.07*	*0.05*
IL1R1 Interleukin 1 receptor, type I	1.25	0.64	0.66	0.53
CHUK Conserved helix-loop-helix ubiquitous kinase	1.25	0.65	0.55	*0.44*
*PGK1 Phosphoglycerate kinase 1*	1.23	0.96	0.92	0.75
BCL10 B-cell CLL/lymphoma 10	1.23	*0.25*	*0.26*	*0.21*
*B2M Beta-2-microglobulin*	1.21	0.94	1.01	0.83
HMOX1 Heme oxygenase (decycling) 1	1.17	**6.73**	**6.92**	**5.90**
FADD Fas (TNFRSF6)-associated via death domain	1.09	*0.08*	*0.04*	*0.04*
*PPIA Peptidylprolyl isomerase A*	1.09	0.97	1.04	0.95
*GUSB Glucuronidase, beta*	1.08	0.92	0.72	0.66
ATF1 Activating transcription factor 1	1.06	0.67	0.62	0.58
*ACTB Actin, beta*	1.06	1.08	0.88	0.83
IFNAB1 Interferon, beta 1	0.98	*0.11*	*0.41*	*0.41*
F2R Coagulation factor II (thrombin) receptor	0.97	1.41	1.40	1.45
PPM1A Protein phosphatase 1A (formerly 2C), alpha isoform	0.93	0.65	0.64	0.69
LTA Lymphotoxin alpha (TNF superfamily, member 1)	0.91	**2.58**	0.91	1.00
GJA1 Gap junction protein, alpha 1, 43kDa	0.90	1.06	0.80	0.88
NOD1 Nucleotide-binding oligomerization domain containing 1	0.90	*0.11*	*0.35*	*0.39*
LTBR Lymphotoxin beta receptor	0.88	0.84	0.75	0.85
TRADD TNFRSF1A-associated via death domain	0.87	*0.41*	*0.50*	0.58
*HPRT1 Hypoxanthine phosphoribosyltransferase 1*	0.79	0.76	1.10	1.39
EGR1 Early growth response 1	0.66	1.16	**2.23**	**3.41**
JUN Jun oncogene	0.64	*0.12*	*0.22*	*0.34*
MAP3K1 Mitogen-activated protein kinase kinase kinase 1	0.60	*0.16*	*0.10*	*0.17*
MALT1 Mucosa associated lymphoid tissue lymphoma translocation gene 1	0.55	0.62	0.60	1.09
TNF Tumor necrosis factor (TNF superfamily, member 2)	0.55	*0.16*	1.79	**3.27**
IKBKG Inhibitor of kappa light polypeptide gene enhancer in B-cells, kinase gamma	*0.43*	0.92	0.43	0.99
TLR3 Toll-like receptor 3	*0.41*	*0.08*	*0.25*	0.61
FASLG Fas ligand (TNF superfamily, member 6)	*0.13*	*0.00*	*0.00*	*0.01*
SELP Selectin P	*0.02*	*0.10*	*0.12*	7.41

qRT-PCR was performed using the NF-κB primer library (RealTimePrimers, Elkins Park, PA, USA).

See Material and Methods section for details. The gene expression levels were normalized to the expression of ribosomal protein 13a (RPL13a). The Curcumin effect on IL-1β-induced gene expression was calculated as ratio of the gene expression in cells treated with IL-1β+Curcumin and cells treated with IL-1β alone. Relative changes higher than 2-fold are marked bold. Relative changes lower than 0.5-fold are marked italic. Putative housekeeping genes other than RPL13a are indicated by italics.

In response to Curcumin, we detected 10 induced genes, but 24 repressed genes. 7 of the induced genes were also induced by IL-1β. From the genes repressed by Curcumin, 9 were induced and 3 were repressed by IL-1β.

Cotreatment with IL-1β and Curcumin induced 12 genes, whereas 16 genes were repressed. 10 of the induced genes were also induced by IL-1β alone. From the genes repressed by cotreatment with IL-1β and Curcumin, 1 was induced and 4 were repressed by IL-1β.

**Figure 9:**
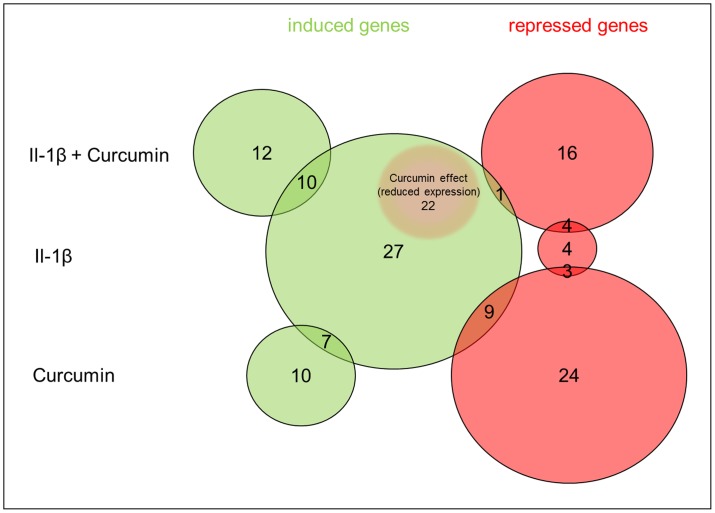
Illustration of the data presented in table 1, representing the number of genes induced (green) or repressed (red) by treatment with IL-1β, Curcumin or IL-1β+Curcumin. The size of the circles is proportional to the number of genes in each group. The threshold was set over 2 and under 0.5-fold variation in gene expression. Additionally, the Curcumin effect indicating reduced expression is indicated. Numbers in overlaps indicate the number of genes that shared in the groups.

Curcumin was able to reduce or even abolish IL-1β induced expression of 22 genes. The strongest inhibitory Curcumin effect, which was calculated from the ratio of the gene expression in cells treated with IL-1β+Curcumin and cells treated with IL-1β alone, was observed for IL-1β expression. The results are illustrated in Fig. 9.

## Discussion

IL-1 signaling plays an important role in malignant tumors. Increased local expression of IL-1 has been correlated with tumor invasiveness and poor prognosis in cancer patients [2]. Upregulated IL-1 expression has been described in several tumors, including breast, colon, lung, head and neck cancers, and melanomas [13]. The function of the IL-1R agonistic proteins IL-1α and IL-1β, as well as the naturally occurring IL-1R antagonist (IL-1Ra) has been studied in knockout (KO) mice. As shown by Voronov et al. [3] IL-1β is required for tumorigenicity of B16 melanoma cells and angiogenesis. Impaired tumorigenicity was described for 3-methylcholanthrene-induced tumors in IL-1β KO mice, whereas most rapid tumor development and inflammation was demonstrated in IL-1Ra KO mice [14]. Sustained treatment with sufficient levels of IL-1Ra was shown to reduce tumor growth, vascularization, and the number of lung metastases in melanoma-bearing mice [15]. However, membrane-associated IL-1α expression in fibrosarcoma cells was shown to reduce tumorigenicity by inducing antitumor immunity in wildtype mice [16]. Hence, the treatment of cancers with IL-1 blockade, particularly by blocking IL-1β activity, appears to be a promising strategy, especially in addition to antiangiogenic therapies [17].

In our previous studies we showed that IL-1β treatment leads to increased VEGF-A expression in C3842 chondrosarcoma cells [8], [18]. In this study we additionally demonstrated IL-1β-induced VEGF-A expression in SW1353 chondrosarcoma cells. These results imply that the regulatory pathway is intact and VEGF-A is not constitutively expressed in chondrosarcoma cells. Besides VEGF-A several other genes may be regulated by IL-1β. Vincenti and Brinckerhoff [19] identified several IL-1β regulated early response genes in SW1353 cells using microarray analysis, including transcription factors, cytokines, growth factors and their receptors, proteases, matrix proteins, adhesion molecules, signaling intermediates and tumor suppressors. Regarding transcription factors they showed that members of the NF-κB family, the AP-1 family and the ETS-family were modulated by IL-1β. Gebauer et al. [20] confirmed the activation of NF-κB target genes by IL-1β in SW1353 cells. Therefore, IL-1 signaling is obviously an important pathway in chondrosarcoma cells.

We demonstrated IL-1 signal transduction in C3842 and SW1353 cells by the detection of IL-1β induced IκBα phosphorylation, which represents the essential downstream event in NF-κB activation. As reviewed by Kawai and Akira [6], IκBα interacts with NF-κB and keeps it as a latent and inactive form in the cytoplasm, until IκBα is phosphorylated, ubiquitinated and degraded by the 26S proteasome, allowing NF-κB to translocate into the nucleus. Accordingly, we detected the disappearance of phosphorylated IκBα due to degradation in C3842 and SW1353 cells treated with IL-1β, indicating NF-κB activation. Additionally, we demonstrated IL-1β-induced nuclear translocation of NF-κB in C3842 and SW1353 cells. IL-1β induced activation and nuclear translocation of NF-κB was previously also shown in normal articular chondrocytes [21].

IL-1 blocking agents such as anakinra (recombinant IL-1Ra), canakinumab (a monoclonal anti-IL-1β antibody) or rilonacept (a fusion protein of the two extracellular chains of the IL-1R complex linked to the Fc segment of IgG) are used in various chronic and inflammatory human diseases [17]. Regarding bone and joint diseases anakinra has been approved for the therapy of rheumatoid arthritis (RA). The clinical efficacy of anakinra in RA was demonstrated in several placebo-controlled trials, where anakinra improved the signs and symptoms of RA and retarded the rate of structural joint damage [22]. To our knowledge neither of these IL-1 blocking agents has been tested in the treatment of cartilage tumors. Certainly, IL-1 signaling may be prevented by any of these agents in chondrosarcoma cells. The results of our study account for investigations on the efficacy of IL-1 blocking agents in chondrosarcomas, especially regarding the selective blockade of IL-1β by canakinumab.

In contrast to the aforementioned IL-1 blocking agents Curcumin blocks IL-1 signal transduction very early by preventing the recruitment of IRAK to IL-1R, which inhibits downstream events in the IL-1 signal cascade [11]. Accordingly, we demonstrated that IL-1β induced IκBα phosphorylation, which leads to NF-κB activation by nuclear translocation, was blocked in C3842 and SW1353 cells by Curcumin treatment. We defined an appropriate Curcumin concentration and incubation time prior to IL-1β treatment to block IL-1 signaling in chondrosarcoma cells. The blockade of IL-1β induced NF-κB activation by Curcumin was previously also described in articular chondrocytes [23], [24]. Recently, it was demonstrated that Curcumin inhibits IL-1α-induced aggrecan loss in articular cartilage [25]. Hence, Curcumin is suitable to block IL-1 signaling in cartilaginous cells, including chondrosarcoma cells.

The activation of NF-κB leads to the expression of numerous genes that are involved in cell survival, proliferation, invasion, angiogenesis, and metastasis [26]. Among NF-κB regulated proangiogenic factors is VEGF-A, which is the most important mediator of angiogenesis, and is overexpressed by a multitude of solid human tumors [27]. Previously we reported on the significance of VEGF-A expression in conventional chondrosarcomas and its correlation with the proliferating capillary index [8], [9]. We demonstrated that VEGF-A expression is regulated by IL-1β in chondrosarcoma cells and assumed that IL-1 blockade may be a therapeutic option in chondrosarcoma. As expected from our experiments on IκBα phosphorylation and nuclear translocation of NF-κB we demonstrated that Curcumin blocks IL-1β-induced VEGF-A expression in C3842 and SW1353 cells. Additionally, we showed that IL-1β induced angiogenesis by cell culture supernatants of chondrosarcoma cells was blocked by Curcumin using an in vitro tube forming assay. To our knowledge, we are the first to show these effects in chondosarcoma cells. At least, similar effects of Curcumin on VEGF-A expression were also reported in human articular chondrocytes [28]. Moreover Csaki et al. [28] demonstrated that Curcumin inhibits further IL-1β induced, NF-κB-dependent mediators in chondrocytes, such as Cox-2, MMP-3 and MMP-9.

To further analyse the effects of IL-1 blockade on NF-κB-related gene expression in chondrosarcoma cells, we used gene expression profiling. Because the data presented here do only represent a screening experiment, the quantification must be interpreted carefully. However, the experiment clearly showed that IL-1β activated several NF-κB related genes and that Curcumin was able to reduce the mRNA expression of most of these IL-1β activated genes. But there were also some genes that were activated by Curcumin alone and by combined treatment with Curcumin and IL-1β. This seems not surprising, as Curcumin has also been described to influence other signaling pathways than NF-κB [29], [30].

An inhibition of the expression of many of these genes might be beneficial as there is a common relation to tumor biology, which might contribute to the anti-tumor activity of Curcumin. A significant number of IL-1β induced genes are cytokines, such as Colony stimulating factor 2 (CSF2) also known as granulocyte/macrophage colony-stimulating factor (GM-CSF), Chemokine (C-C motif) ligand 2 (CCL2) and IL-6. Also IL-1β and IL-1α were induced by IL-1β treatment, showing a feed-forward effect and demonstrating that stimulated chondrosarcoma cells are able to produce these cytokines. The results also demonstrated IL-1β-induced up-regulation of tumor necrosis factor (TNF)-pathway. 6 members of the TNF receptor superfamily were up-regulated, whereas the expression of TNF-related signaling molecules was to some extent down regulated. TNF-α is known to modulate the expression of matrix metalloproteinases (MMPs), which are modulators of cell motility and invasion [31]. Up-regulation of MMP-1 and MMP-13 by IL-1β was previously demonstrated in chondrosarcoma cells [20]. IL-1β-induced up-regulation of CD40 molecule, TNF receptor superfamily member 5 was also reported in human articular chondrocytes [19].

About CSF2, which was the most up-regulated gene by IL-1β, no reports on chondrosarcomas have been published to our knowledge so far. However, it was reported that this molecule is important for osteoclast activation by bone metastases in breast cancer [32] and for the mediation of bone cancer pain [33]. Inhibiting GM-CSF signaling was reported to reduce tumor growth [33], but earlier studies also demonstrated its function in macrophage tumoricidal activity [34].

The third most up-regulated gene after IL-1β was ICAM1, which is a cell adhesion molecule that was recently associated with cell motility and migration of chondrosarcoma cells [35]. A similar effect in chondrosarcoma cells was described for CCL2, also known as monocyte chemoattractant protein (MCP)-1 [36]. IL-1β-induced up-regulation of IL-6 was previously described in human articular chondrocytes and chondrosarcoma cells [20].

Although Curcumin was shown to be appropriate to block IL-1β-induced gene expression in cultured cells, its therapeutic use is generally limited due to poor absorption, rapid metabolism and elimination [37]. Moreover, the tissue distribution of Curcumin is obviously variable as shown in mice after intraperitoneal administration [38]. To our knowledge the bioavailability of Curcumin was not determined, neither in cartilage nor in cartilage tumors. We assume that the bioavailability of Curcumin in cartilaginous tissues may be low after oral, intraperitoneal or intravenous administration. Further attempts to enhance the bioavailability of Curcumin are necessary, such as the combination with adjuvants, the use of nanoparticles, liposomes, micelles, phospholipid complexes or bioconjugates [37]. Also derivatives of Curcumin, such as EF-24, may be promising substances [39].

As Curcumin does not selectively block microenvironment-derived IL-1β signaling, but also membrane-associated IL-1α signaling, which has been linked with antitumor immunity [16], the use of Curcumin or its dervatives should be considered carefully, depending on the therapeutic strategy and the individual tumor. We assume that Curcumin or Curcumin derivatives are appropriate to block IL-1 signaling also in chondrosarcomas, if an adequate bioavailability is achieved in the tumor tissue. Therefore, Curcumin should be tested in an appropriate tumor model and compared with agents specifically targeting IL-1β signaling, such as canakinumab. The remaining question is: Why not treat human cancers with IL-1 blockade?
